# Effects of an Ecosystem Engineer on Belowground Movement of Microarthropods

**DOI:** 10.1371/journal.pone.0062796

**Published:** 2013-04-30

**Authors:** Erin K. Cameron, Heather C. Proctor, Erin M. Bayne

**Affiliations:** Department of Biological Sciences, University of Alberta, Edmonton, Alberta, Canada; Estacion Experimental de Zonas Áridas (CSIC), Spain

## Abstract

Ecosystem engineers affect other species by changing physical environments. Such changes may influence movement of organisms, particularly belowground where soil permeability can restrict dispersal. We investigated whether earthworms, iconic ecosystem engineers, influence microarthropod movement. Our experiment tested whether movement is affected by tunnels (i.e., burrows), earthworm excreta (mucus, castings), or earthworms themselves. Earthworm burrows form tunnel networks that may facilitate movement. This effect may be enhanced by excreta, which could provide resources for microarthropods moving along the network. Earthworms may also promote movement via phoresy. Conversely, negative effects could occur if earthworms alter predator-prey relationships or change competitive interactions between microarthropods. We used microcosms consisting of a box connecting a “source” container in which microarthropods were present and a “destination” container filled with autoclaved soil. Treatments were set up within the boxes, which also contained autoclaved soil, as follows: 1) control with no burrows; 2) artificial burrows with no excreta; 3) abandoned burrows with excreta but no earthworms; and 4) earthworms (*Lumbricus rubellus*) present in burrows. Half of the replicates were sampled once after eight days, while the other half were sampled repeatedly to examine movement over time. Rather than performing classical pairwise comparisons to test our hypotheses, we used AIC_c_ to assess support for three competing models (presence of tunnels, excreta, and earthworms). More individuals of Collembola, Mesostigmata, and all microarthropods together dispersed when tunnels were present. Models that included excreta and earthworms were less well supported. Total numbers of dispersing Oribatida and Prostigmata+Astigmata were not well explained by any models tested. Further research is needed to examine the impact of soil structure and ecosystem engineering on movement belowground, as the substantial increase in movement of some microarthropods when corridors were present suggests these factors can strongly affect colonization and community assembly.

## Introduction

The ability of ecosystem engineers to modify their physical environment means that they often have strong effects on other organisms [1], [2]. Changes in habitat structure due to ecosystem engineering can influence resource availability, species’ abundances, and community composition [1]–[3]. Movement of organisms is another key factor that may be affected by engineers, although it has been poorly studied. For example, post-larval dispersal of some macrofauna is greater in beds of the mussel *Mytilus edulis* L. as compared to in bare sediment patches, where turbulence and sediment flux are lower [4]. Modeling suggests that ecosystem engineers can even cause increases in their own rate of spread because of their ability to alter habitat structure [5]. They may also decrease movement, as in the case of shrubs in Mediterranean woodland that act as a physical obstacle to seed dispersal and thereby affect herbaceous plant communities [6].

The effects of ecosystem engineering on movement may be particularly important belowground, where permeability of the soil matrix limits the active movement of many groups [7], [8]. Belowground movement is estimated to be approximately four times slower than aboveground locomotion for some microarthropods [9]. However, burrowing by soil macrofauna can increase porosity and create openings that might facilitate the movement of other organisms [7].

Earthworms are frequently cited examples of belowground ecosystem engineers because of their large impacts on soil structure, which can lead to cascading effects on other species, including songbirds [10], amphibians [11], [12], and plants [13]. For example, earthworms and their burrowing activity can facilitate the movement and germination of giant ragweed (*Ambrosia trifida* L.) seeds [14]. Effects of earthworms vary depending on whether they dwell in litter (epigeic), in mineral soil (endogeic), or in deep burrows while feeding on leaf litter (anecic) [15]. Their impacts may be especially substantial in ecosystems where they are exotic and there are no native earthworms, as is the case in most of Canada and the northern United States [16]–[18].

In their native and introduced ranges, earthworms have been observed to significantly affect soil microarthropods [19]–[22]. Both positive and negative effects on microarthropod abundance and species richness have been reported. Impacts seem to depend on the density of earthworms, to what ecological group they belong, and the microarthropod taxa being examined. For example, microarthropods that are mainly detritivorous or microbivorous (Collembola, Astigmata, Oribatida) have been suggested to be more negatively affected than the predatory Mesostigmata due to competition or to greater disruption of their food resources by earthworms [22], [23]. Earthworms can also have variable effects on microarthropod distributions due to their effects on physicochemical properties of soil, with microarthropods being attracted to earthworms and their excreta in some cases [24]–[26] but not in others [25]–[27]. These responses appear to vary depending on the earthworm species and microarthropod species involved [26].

One of the ways in which earthworms are thought to affect microarthropods is by altering their rate of movement [22], [23], [28]. Some microarthropods can disperse aboveground via wind, water, phoresy, or walking [29], but their movement belowground has been less well studied. Earthworms may influence microarthropod movement via a number of mechanisms. Firstly, earthworm burrows have been suggested to act as corridors for movement of microarthropods by forming an interconnected network of pores within the soil that are easier to move through [22], [23], [28]. Secondly, microarthropods may be attracted to burrows by the earthworm secretions and excreta (mucus, castings, and urine) that line burrow walls [24], [28] and may subsequently move through the burrows in the pursuit of microbial food resources present there [24]. Thirdly, earthworms themselves may transport microarthropods via phoresy, as appears to occur with nematodes [30], [31]. Finally, earthworms could consume some microarthropod taxa or negatively affect them via disturbance of the soil or competition for microbial food resources, thereby causing a reduction in movement and abundance [20], [22], [32].

Here we examine the effects of an epi-endogeic earthworm (*Lumbricus rubellus* Hoffmeister, 1843) on movement of microarthropods in boreal forest soil using a microcosm experiment. *Lumbricus rubellus* lives and feeds in the upper soil layers and is a common invader in Canada and many other countries [33], [34]. Our experiment tested whether movement of springtails and mites is affected by the presence of tunnels in the soil, of tunnels lined with excreta, or of earthworms themselves. We predicted microarthropod movement would be greater when there were tunnels in the soil, particularly for Mesostigmata and Collembola, which typically have a larger body size than other microarthropod taxa and hence may be more limited by soil porosity than smaller-bodied taxa [35], [36]. Variation in the impacts of earthworms on movement of microarthropod taxa is one factor that could explain changes in community composition of microarthropods in response to earthworms reported in previous studies.

## Materials and Methods

### Ethics Statement

No specific permits were required for the described field studies, as the location where samples were collected is not privately-owned or protected. The field studies did not involve endangered or protected species. Data collected in this study will be available at https://era.library.ualberta.ca/public/home.

### Experimental Design

**Figure 1:**
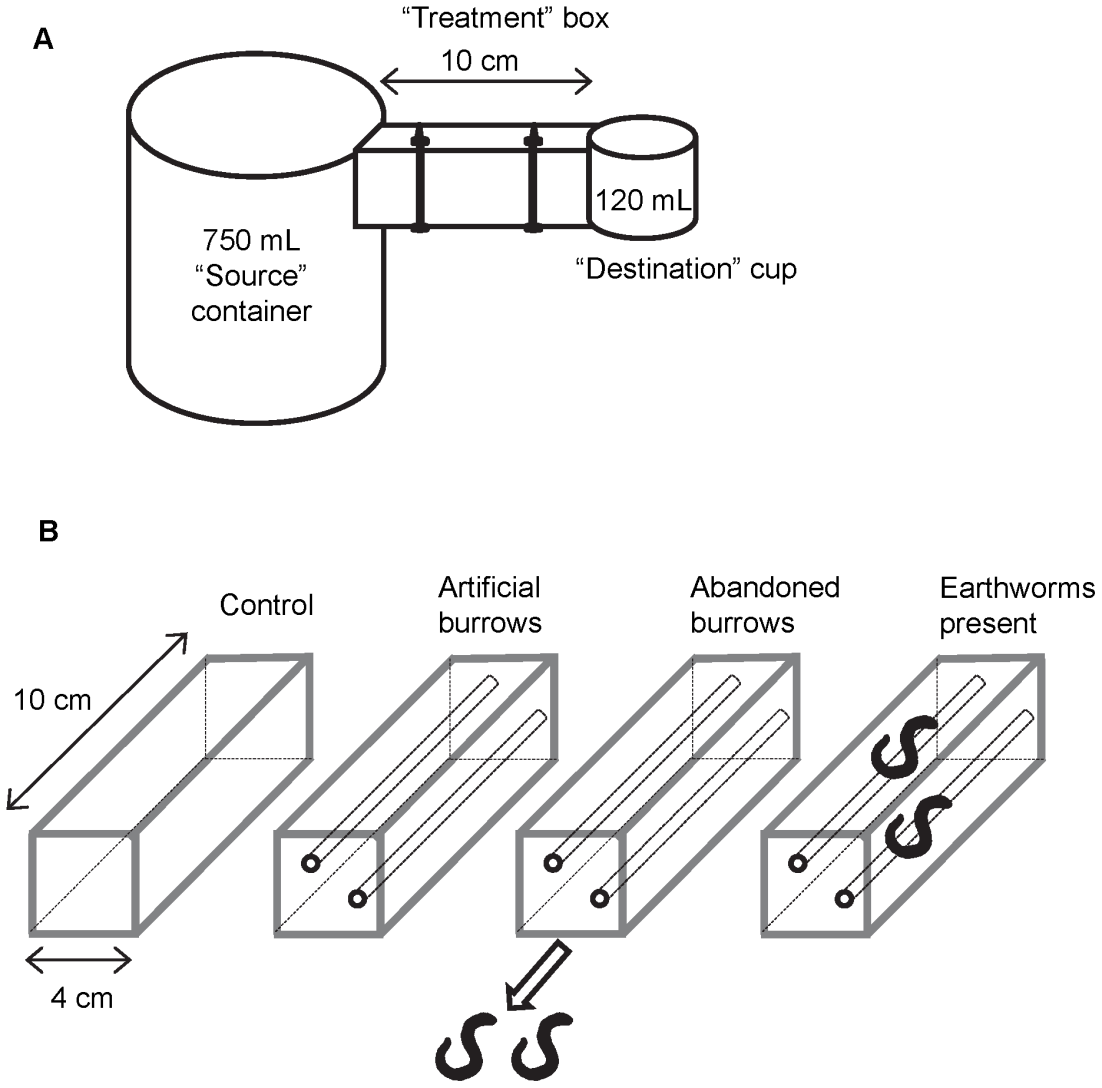
Experimental set-up. (a) A microcosm consisting of a 750 mL “source” container, a 10 cm long “treatment” box in which the treatments were implemented, and a 120 mL “destination” container; (b) The four treatments within the boxes, including the control treatment with no earthworms, the artificial burrows treatment with two tunnels made by a dowel, the abandoned burrows treatment in which earthworms were removed before the experiment, and the earthworms present treatment in which earthworms were present throughout the experiment.

We used a short-term microcosm experiment to investigate the effects of non-native earthworms on microarthropod movement. Each microcosm consisted of an open-ended 10 cm×4 cm×4 cm box with one 750 mL (∼12.5 cm height×9 cm diameter) opaque plastic container and one 120 mL (∼5 cm height×5.5 cm diameter) translucent plastic container attached to either end (Figure 1a). The 750 mL container acted as a “source” of microarthropods and the 120 mL container as a “destination”. The sides of the plastic containers were cut where the box was attached to allow movement of microarthropods and earthworms across the microcosm. The bottom and sides of each box were constructed from 0.6 cm thick plywood, while the top was 0.3 cm thick acrylic plastic. We drilled two 0.5 cm diameter screw holes in the bottom and top of each box and bolted the top down after the box was filled with soil to limit movement of microarthropods on the soil surface.

Soil was collected from the organic horizon (H layer) of a trembling aspen (*Populus tremuloides* Michx.)/balsam poplar (*Populus balsamifera* L.) forest in the North Saskatchewan River valley in Edmonton, Alberta (53°32′ N 113°33′ W). Earthworms were also collected from this forested area. The soil used to fill the boxes and 120 mL destination containers was sterilized by autoclaving for 1 hour at 121°C. We mixed unsterilized soil and distilled water and sieved the slurry through a 36 µm sieve to obtain water containing microbes but no microarthropods. After allowing the autoclaved soil to cool, the sieved liquid was added to it as a food source for the earthworms and microarthropods. The 750 mL containers were filled with unsterilized soil and acted as the microarthropod source.

Two days before the start of the experiment, we filled the unattached boxes with sterilized soil and set up the treatments within them (Figure 1b). Our experiment included four treatments: 1) a “control” treatment with no earthworms and no tunnels; 2) an “artificial burrow” treatment with no earthworms but two tunnels running the length of the box made by a 0.6 cm thick wooden dowel; 3) an “abandoned burrow” treatment in which two tunnels were made using the dowel and one earthworm was introduced into each tunnel for the 30 hour period immediately prior to the experiment to lay down excreta, and then removed; and 4) an “earthworms present” treatment in which earthworms were introduced into two dowel-made tunnels 30 hours before the experiment began and remained in the microcosms during the experiment. The width of the dowel was approximately equal to the body width of the *L. rubellus* individuals used in the experiment. Although our dowel-made tunnels likely did not mimic the sinuosity of real earthworm burrows, we chose to create the tunnels in this way for all treatments in order to ensure that they were similar lengths across all replicates. For all treatments, the ends of the boxes were capped with aluminum foil that was secured with elastic bands until the microcosms were assembled. Earthworms were washed with distilled water before being added to the boxes. For the abandoned burrow and earthworms present treatments, the earthworms were removed from the boxes after 30 hours by shining a bright light at one end of the box and placing a dark cloth over the other end or by using electric shock as needed. The electric shock was administered to one end of the box using wall current. In the earthworms present treatment, the earthworms were then placed into the source container that was attached to the same box they had been removed from. This was done to test whether microarthropods might travel from the source container to the 120 mL destination container using the earthworms as phoretic hosts. Nothing was used to attract earthworms to the destination containers but, when we removed the containers for sampling, an earthworm was present in the destination container in nine of the twenty-four microcosms in the earthworm treatment.

The experiment was carried out over eight days in October 2011 in a growth chamber located at the University of Alberta with settings of 18°C, relative humidity of 40%, and day length of 14 hours. The eight day time period was chosen to reduce the possibility that reproduction of microarthropods would occur in the destination containers and affect abundances, as we were interested primarily in movement. We randomly assigned the source containers to the treatment boxes and placed the microcosms randomly within the growth chamber. Pin holes were made in the lids of the plastic containers for ventilation, as the lids were closed to prevent escape of microarthropods and earthworms. All microcosms were misted daily to maintain moist conditions.

### Microarthropod Sampling

We examined both the total number of microarthropods that dispersed by the end of the experiment and the cumulative movement of microarthropods over time. Each of the four treatments had 24 replicate microcosms, with 12 replicates sampled only at the end of the experiment and 12 replicates sampled at multiple times to investigate movement over time. At each sampling time, we extracted microarthropods from the soil in the destination containers (extraction method described below). For the replicates in which the 120 mL destination container was not removed until the end of the experiment (192 hours), there were a total of 48 extractions (4 treatments×12 replicates). This provided cumulative numbers of dispersers over 8 days. For the replicates sampled at multiple times, the destination container was removed from the source container at 4 h, 12 h, 24 h, 48 h, 96 h, and 192 h after the start of the experiment and immediately replaced with a new destination container filled with autoclaved soil (except following removal of the 192 h container at the end of the experiment). Thus, for example, the destination containers removed at 192 h would have had 96 h between the time of their attachment and removal to accumulate dispersing microarthropods, since the previous samples were removed at 96 h. A total of 288 extractions (4 treatments×12 replicates×6 time steps) were performed for this analysis and these repeated samples were used to determine whether there was an effect of treatment on rates of movement over time.

At each time step, we also extracted microarthropods from an additional 120 mL sample of autoclaved soil that was not attached to a box or source container to verify that the autoclaving had indeed sterilized the soil of microarthropods. At the end of the experiment, we extracted a 120 ml sample from each of the 96 750 mL source containers.

Microarthropods were live-extracted at the Royal Alberta Museum in Edmonton using Tullgren funnels [37]. All extractions ran for 7 days and microarthropods were preserved in 80% ethanol. We sorted microarthropods into non-astigmatan Oribatida, Astigmata, Prostigmata, Mesostigmata, and Collembola. Because there were low numbers of similarly sized (very small) Prostigmata and Astigmata, they were grouped together for statistical analyses. We did not identify the mites to finer taxonomic levels because there was inconsistent taxonomic representation among the samples. The majority of Collembola belonged to the Onychiuridae, although representatives of Entomobryidae, Isotomidae, and Hypogastruridae were present as well.

### Statistical Analyses

We assessed the effects of our treatments on the total number of microarthropods dispersing into the destination container using Poisson or negative binomial regression in Stata 9.1 (Stata-Corp, College Station, Texas) depending on the distribution that best fit the data for each group. Negative binomial regression was used when data were overdispersed. Analyses were conducted for Collembola, Mesostigmata, Oribatida, Prostigmata+Astigmata, and all microarthropods together. There were 24 replicates per treatment in all of the models examined, for a total sample size of 96. In 12 of the replicates in each treatment, microarthropods were extracted at six time steps (4 h, 12 h, 24 h, 48 h, 96 h, 192 h) and the total number of microarthropods across all time steps was used in the analysis. Because microarthropod extraction occurred only at the end of the experiment in the remaining 12 replicates per treatment, all models included a variable to account for whether multiple or single extractions were performed for a given replicate microcosm.

We used dummy variable coding to re-categorize our four treatments into groups and AIC_c_ (Akaike’s information criterion, corrected for small sample sizes) to assess support for three competing models explaining microarthropod abundance in the destination containers (see Supporting Materials S1 for an example of dummy variable coding). This approach is analogous to the use of planned comparisons within an ANOVA to test specific hypotheses [38]–[40]. AIC_c_ estimates the relative distance between a model and the mechanism that generated the observed data [41]. It relies on information theory and the principle of parsimony to provide a weight of evidence for different hypotheses. We considered models with ΔAIC_c_<2 to be plausible [41]. The models examined were the presence of tunnels (earthworms present, abandoned burrow, and artificial burrow treatments vs. the control), the presence of earthworms during the experiment (earthworms vs. abandoned burrow, artificial burrow, and control), and the presence of earthworm castings/mucus (earthworms and abandoned burrow vs. artificial burrow and control) (see Supporting Materials S1 for an explanation of model parameters). We compared our three models to a global model which included a “treatment” variable (a categorical variable coding the four treatments) and a null model. All of the models, including the null model, had a variable to control for whether single vs. multiple extractions were performed. As well, negative binomial regression was used to test whether abundances of taxa differed among the source containers.

We also investigated whether our treatments influenced the cumulative total number of microarthropods dispersing over time (i.e., movement rates) using Poisson and negative binomial regression. This separate analysis had a sample size of 48 (12 replicates per treatment) for all models because time series data were only available for the replicates from which multiple samples were taken. A random effect was included in all models to account for the lack of independence between multiple samples taken from the same replicate over time. AIC_c_ was again used to rank three competing models (presence of tunnels, presence of earthworms during the experiment, and presence of earthworm castings/mucus) and to compare them to global and null models. We examined support for these models both with time as an interacting variable and as an independent predictor.

## Results

### Abundance

**Figure 2:**
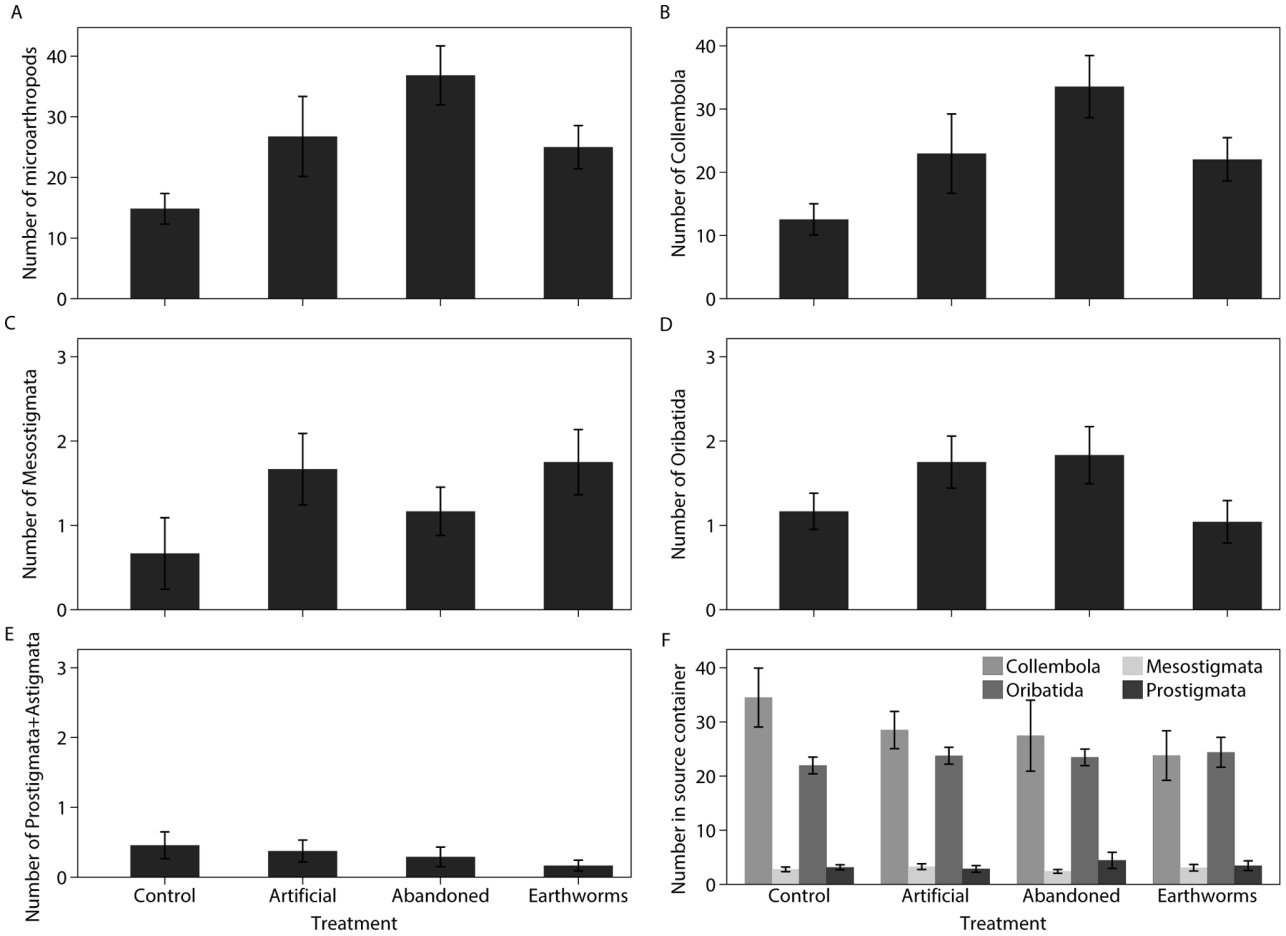
Total number of microarthropods in destination containers at the end of the experiment. (a) All microarthropods together (±SE) in the destination containers; (b) Collembola in the destination containers; (c) Mesostigmata in the destination containers; (d) Oribatida in the destination containers; (e) Prostigmata+Astigmata in the destination containers; and (f) Collembola, Mesostigmata, Oribatida, and Prostigmata+Astigmata in a 120 mL sample from the source containers. N = 24 replicates per treatment.

A total of 2482 microarthropods were extracted from the destination containers, including 2186 Collembola, 126 Mesostigmata, 139 Oribatida, 27 Prostigmata, and 4 Astigmata. No microarthropods were recovered from the autoclaved soil samples that were not attached to source containers, confirming that our destination containers were indeed free of microarthropods at the beginning of the experiment. Oribatida abundance fit a Poisson distribution, while all other taxa showed evidence of overdispersion and therefore negative binomial regression was used. According to the ΔAIC_c_ scores, the tunnel model was the best supported and most parsimonious model for explaining the abundances of Collembola, Mesostigmata and all microarthropods together, although the global model was also well supported for all microarthropods and Collembola (Figure 2a–e; Table 1). For Oribatida abundance, the earthworm presence (ΔAIC_c_ = 0.00), global (ΔAIC_c_ = 0.20), and null models (ΔAIC_c_ = 1.79) received the most support. The null model was the best supported model for Prostigmata+Astigmata, but the worm, castings, and tunnels models also well supported with ΔAIC_c_ scores less than 2. In the source container samples, the abundance of each taxon did not differ significantly across treatments (Figure 2f; Collembola χ^2^ = 1.85, P = 0.604; Mesostigmata χ^2^ = 1.87, P = 0.599; Oribatida χ^2^ = 0.970, P = 0.809; Prostigmata+Astigmata χ^2^ = 2.11, P = 0.551).

**Table 1:** Regression fit statistics for models predicting microarthropod abundance.

Taxa	Model	*K*	LL	ΔAIC_c_	*w*AIC_c_
Microarthropods	**Tunnels**	**4**	**−393.80**	**0.00**	**0.52**
	**Global**	**6**	**−391.68**	**0.26**	**0.45**
	Castings	4	**−**396.85	6.09	0.02
	Null	3	**−**399.67	9.55	0.00
	Worms	4	**−**399.59	11.59	0.00
Collembola	**Tunnels**	**4**	**−387.89**	**0.00**	**0.53**
	**Global**	**6**	**−385.94**	**0.59**	**0.40**
	Castings	4	**−**390.15	4.51	0.06
	Null	3	**−**392.91	7.84	0.01
	Worms	4	**−**392.85	9.92	0.00
Mesostigmata	**Tunnels**	**4**	**−148.57**	**0.00**	**0.53**
	Null	3	**−**150.71	2.11	0.18
	Worms	4	**−**150.09	3.05	0.12
	Global	6	**−**148.04	3.43	0.09
	Castings	4	**−**150.50	3.85	0.08
Oribatida	**Worms**	**3**	**−147.68**	**0.00**	**0.36**
	**Global**	**5**	**−145.58**	**0.20**	**0.32**
	**Null**	**2** **3**	**−149.64**	**1.79**	**0.15**
	Tunnels	3	**−**148.72	2.09	0.13
	Castings	3	**−**149.64	3.91	0.05
Prostigmata+Astigmata	**Null**	**3**	**−68.34**	**0.00**	**0.31**
	**Worms**	**4**	**−67.33**	**0.16**	**0.28**
	**Castings**	**4**	**−67.51**	**0.52**	**0.24**
	**Tunnels**	**4**	**−68.02**	**1.56**	**0.14**
	Global	6	**−**67.14	4.29	0.04

Predictors included presence of tunnels (*Tunnels*), earthworm excreta (*Castings*), and earthworms (*Worms*). All models included a variable to account for whether microarthropods were extracted from a replicate at multiple times versus only once at the end of the experiment. The best model has a ΔAIC_c_ of zero and the highest *w*AIC_c_ value. Models with ΔAIC_c_<2 are also considered to be plausible and are shown in bold. With *k*, number of parameters; LL, log likelihood; ΔAIC_c_, difference in the Akaike’s information criterion (corrected for small sample size) value between model and the most strongly supported model; *w*AIC_c_, weight given by the AIC (i.e., relative strength of support for model). See Supporting Materials S1 for further explanation of parameter numbers and dummy variable coding.

### Cumulative Movement Over Time

**Figure 3:**
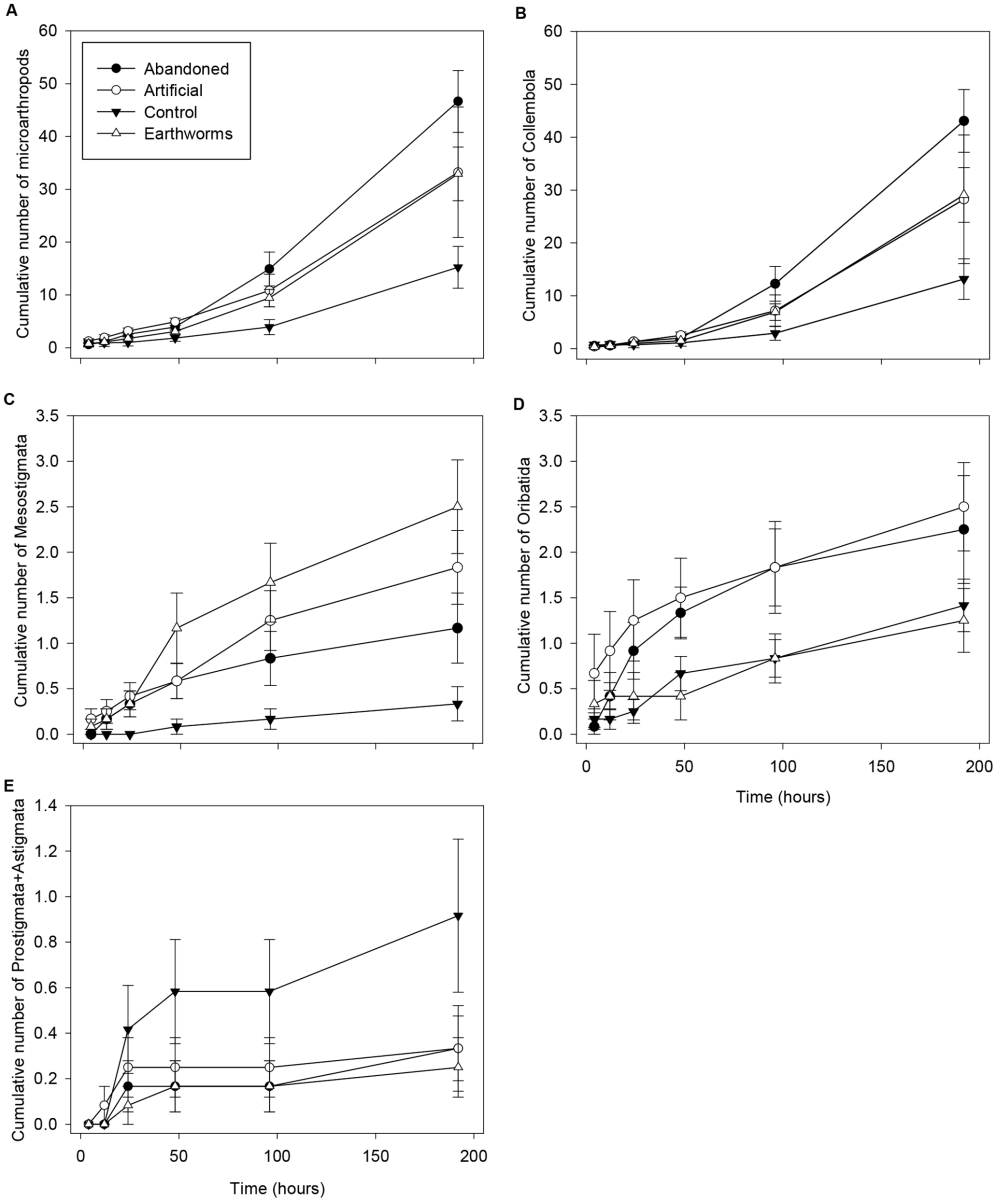
Cumulative number of microarthropods dispersing over time. (a) All microarthopods together (±SE); (b) Collembola; (c) Mesostigmata; (d) Oribatida; and (e) Prostigmata+Astigmata. N = 12 replicates per treatment.

Some individuals crossed the 10 cm treatment box and reached the destination containers within the first time step (4 hours) in the artificial burrow and earthworm present treatments for Mesostigmata and in all treatments for Oribatida and Collembola (Figure 3). Therefore, the maximum movement rate for these taxa was 2.5 cm/hour. The first Prostigmata+Astigmata individuals dispersed within 12 hours of the start of the experiment in the artificial burrow treatment, resulting in a maximum movement rate of 0.83 cm/hour. Movement was slower in the control treatment for Mesostigmata and Prostigmata+Astigmata, with the first individuals arriving within 48 hours and 24 hours, respectively. Oribatida and Mesostigmata abundances were analyzed using Poisson regression and negative binomial regression was used for the remaining taxa. For all microarthropods together, the global model that included interactions between time and each of the other variables (tunnel, earthworm, and excreta) was the best supported model explaining cumulative movement over time (Table 2). The tunnel model with no interaction received the most support for Collembola. For Mesostigmata, the tunnel (ΔAIC_c_ = 0.00), tunnel interaction (ΔAIC_c_ = 1.10), and global (ΔAIC_c_ = 1.92) models were best supported. The tunnel (ΔAIC_c_ = 0.00), global (ΔAIC_c_ = 0.052), tunnel interaction (ΔAIC_c_ = 0.34), and earthworm models (ΔAIC_c_ = 0.42) were the best models for Oribatida. There were too few Prostigmata+Astigmata to allow analysis of their movement over time. Assuming there was no reproduction in the source containers for any of the microarthropod groups, a disproportionately smaller number of Oribatida moved to the destination containers (about 1/10^th^ of the totals per treatment) than for the other groups (about 1/3-1/2 of the totals) (Figure 2f versus Figure 2b–e).

**Table 2:** Regression fit statistics for models of microarthropod abundance over time.

Taxa	Model	*K*	LL	ΔAIC_c_	*w*AIC_c_
Microarthropods	Global*Time	**10**	**−682.19**	**0**	**0.68**
	Tunnels*Time	6	**−**690.02	2.91	0.16
	Tunnels	5	**−**690.30	3.48	0.12
	Global	7	**−**689.62	6.31	0.03
	Castings*Time	6	**−**690.48	8.03	0.01
	Castings	5	**−**696.92	16.73	0.00
	Worms	5	**−**701.08	25.05	0.00
	Worms*Time	6	**−**700.02	27.09	0.00
	Null	3	**−**869.92	358.60	0.00
Collembola	**Tunnels**	**5**	**−579.95**	**0**	**0.49**
	Tunnels*Time	6	**−**579.95	2.08	0.17
	Global*Time	10	**−**575.72	2.12	0.17
	Global	7	**−**578.97	2.22	0.16
	Castings	5	**−**587.58	15.26	0.00
	Worms	5	**−**592.86	25.81	0.00
	Castings*Time	6	**−**584.65	11.49	0.00
	Worms*Time	6	**−**592.13	26.45	0.00
	Null	3	**−**748.20	332.37	0.00
Mesostigmata	**Tunnels**	**4**	**−224.48**	**0**	**0.50**
	Tunnels*Time	**5**	**−224.00**	**1.10**	**0.29**
	**Global**	**6**	**−223.36**	**1.92**	**0.19**
	Global*Time	9	**−**222.71	6.96	0.02
	Worms	4	**−**229.88	10.80	0.00
	Castings	4	**−**230.55	12.14	0.00
	Worms*Time	5	**−**229.81	12.73	0.00
	Castings*Time	5	**−**230.55	14.20	0.00
	Null	2	**−**280.15	107.2406	0.00
Oribatida	**Tunnels**	**4**	**−303.43**	**0**	**0.22**
	**Global**	**6**	**−301.37**	**0.052**	**0.21**
	Tunnels*Time	**5**	**−302.56**	**0.34**	**0.18**
	**Worms**	**4**	**−303.63**	**0.42**	**0.18**
	Castings	4	**−**304.55	2.26	0.07
	Worms*Time	5	**−**303.63	2.48	0.06
	Global*Time	9	**−**299.79	3.23	0.04
	Castings*Time	5	**−**304.42	4.05	0.03
	Null	2	**−**339.18	67.42	0.00

Predictors included presence of openings (*Tunnels*), excreta (*Castings*), and earthworms (*Worms*). Time was included in all models, either on its own or in interaction with the other predictor variables. The best model has a ΔAIC_c_ of zero and the highest *w*AIC_c_ value. Models with ΔAIC_c_<2 are also considered to be plausible and are shown in bold. With *k*, number of parameters; LL, log likelihood; ΔAIC_c_, difference in the Akaike’s information criterion (corrected for small sample size) value between model and the most strongly supported model; *w*AIC_c_, weight given by the AIC (i.e., relative strength of support for model).

## Discussion

Our results suggest that activity of *L. rubellus* earthworms can facilitate microarthropod movement. Artificial tunnels in the soil increased the total number of individuals dispersing for several groups including Collembola, Mesostigmata and all microarthropods together, while the presence of earthworm excreta and earthworms themselves resulted in little additional increase. Cumulative movement over time for all microarthropods together was affected by the presence of tunnels and excreta in addition to *L. rubellus*. Abundances of Oribatida and Prostigmata/Astigmata in the destination containers were not well explained by any of the variables considered, with the null models receiving the most support. However, the rate of accumulation of Oribatida over time was influenced by both tunnels and earthworms. Movement of oribatid mites was greater in the presence of tunnels, while presence of earthworms was associated with reduced movement.

Although little is known about belowground dispersal of microarthropods, corridors have been demonstrated to facilitate aboveground dispersal in moss microcosms [42], [43]. Increased movement of microarthropods occurred when moss patches were connected by thin corridors of moss, particularly when the rate of emigration to the hostile surrounding matrix is low (i.e., when movement is biased to occur along the moss corridors) [44]. Our results are also consistent with several studies in which microarthropod abundance increased with soil pore volume [36], [45], [46]. Increased access to resources and reduced predation or competition due to greater availability of refuges have been suggested as mechanisms by which soil pore volume could lead to increased microarthropod abundances [46]. The greater soil heterogeneity created by the tunnels in our experiment might additionally contribute to increases in diversity [47].

The maximum rates of movement observed in our study, even within the control treatment, are much higher than previously recorded for microarthropods within soil in the field [48], [49]. The highest potential rate of movement recorded previously was approximately 20 cm per week for some genera of Oribatida and Collembola [49], in contrast to our maximum estimated rates of 2.5 cm per hour ( = 420 cm/week) for Collembola, Mesostigmata, and Oribatida and 0.83 cm per hour for Prostigmata/Astigmata ( = 210 cm/week). Converting from hours to weeks undoubtedly leads to overestimation of spread rates as it is unlikely that microarthropods would continue to move in the same direction for a week. The fact that movement could only occur along the box also may have resulted in greater estimates of movement speed than would be the case in a natural system, where movement could occur in any direction. The straightness and artificial nature of the tunnels might also have resulted in greater movement rates than would be observed along burrows. Additionally, soil type can have large impacts on movement rates in nematodes [31]. If soil type has similar effects on microarthropod movement, it could account for some variation among studies. Nonetheless, the substantially greater rates of movement in our experiment suggest maximum movement rates may be much larger than previously estimated when movement is highly directional.

Microarthropod taxa can exhibit differing responses to soil pores or corridors [46]. As predicted, artificial tunnels had a stronger effect on movement of Mesostigmata and Collembola which tend to have larger body sizes than Oribatida, Prostigmata, and Astigmata [35], [36]. Large soil pores may be particularly beneficial for taxa that are larger and cannot move as easily through the soil matrix [36]. These varying effects on movement of different microarthropod groups may result in altered predator-prey dynamics or competition among microarthropods and could ultimately lead to shifts in community assembly. We observed that a disproportionately smaller number of Oribatida moved to the destination containers than did the other groups (Fig. 2f versus 2b–e), resulting in a different composition of taxa in the destination containers than in the source containers; however, the treatments themselves did not seem to have a strong influence on the relative proportions of the different taxa remaining in the source containers. A more detailed analysis would be necessary to assess whether other traits besides body size (e.g., diet) can influence the responses of microarthropod taxa to tunnels.

Our artificial and abandoned burrow treatments were designed to mimic the physical changes that occur in the structure of the soil due to earthworm burrowing but not the ongoing disturbance of the soil caused by earthworms burrowing continually. Continual disturbances can be a key factor structuring microarthropod communities, and oribatid mite populations are thought to be particularly sensitive [50], [51]. For example, repeated sieving and mixing of litter and soil resulted in declines in densities of most groups of oribatid mites, as well as Collembola, in a beech forest [51]. It was therefore suggested that high densities of microarthropods in some soils could be related to less mechanical disturbance by earthworms [51]. Although we found a lower cumulative number of Oribatida dispersing over time when *L. rubellus* were present in the microcosms, the presence of earthworms did not have a strong effect on the abundance of any taxa in the destination containers. However, the short duration of our experiment and the disruption of the soil during transport and construction of our microcosms may have reduced our ability to detect negative effects of earthworms per se. How earthworm effects on movement rates influence microarthropod communities over the long-term warrants further investigation.

Our study suggests that the effects of ecosystem engineers on habitat structure may strongly affect movement of other species. Movement of microarthropods was greatest when artificial tunnels were present in the soil, indicating that changes in soil structure, rather than phoresy or changes in nutrient distribution, may influence microarthropod movement belowground. The stronger influence of *L. rubellus* on movement of Collembola and Mesostigmata, as compared to Oribatida, Prostigmata, and Astigmata, suggests that alteration of movement routes is a possible mechanism driving the variable responses of different microarthropod taxa to earthworms. Little is known about movement belowground and the effects of edges and corridors on soil organisms, despite the extensive amount of research in this area in aboveground systems. Consequently, future research should further examine effects of soil structure on soil organisms, including the impacts of other earthworm species on microarthropods.

## Supporting Information

Supporting Materials S1 (DOCX)
