# Supplementary material for: Effects of an Ecosystem Engineer on Belowground Movement of Microarthropods
Source: PLoS One. 2013 Apr 30;8(4):e62796. doi: 10.1371/journal.pone.0062796 (PMC3640026; doi:10.1371/journal.pone.0062796)
Supplement: Supporting Materials S1 — (DOCX) [file pone.0062796.s001.docx]

**Supplemental materials S1**

**Stata code for model contrasts - Example for negative binomial regression of Mesostigmata abundance**

nbreg meso samplingtimes //*nbreg is the command for negative binomial regression

*samplingtimes variable is included in all models as a control for single vs. multiple samples

est store null //*storing the results for the null model

nbreg meso tunnels samplingtimes

est store tunnel //*storing results for the tunnels model

nbreg meso castings samplingtimes

est store castings //*storing results for the castings model

nbreg meso worm samplingtimes

est store worm //*storing results for the worm model

xi: nbreg meso i.treatment //*xi is used with categorical variables

est store global //*stores the global model

est stat * //*exports AIC table for all models

**Example of dummy variable coding in data table**

| ID | treatment | tunnels | castings | worms | samplingtimes | Mesostigmata |
| --- | --- | --- | --- | --- | --- | --- |
| A1 | abandoned | 1 | 1 | 0 | 1 | 0 |
| F1 | artificial | 1 | 0 | 0 | 1 | 1 |
| W1 | worms | 1 | 1 | 1 | 1 | 0 |
| C1 | control | 0 | 0 | 0 | 1 | 5 |
| … to n=96 | … | … | … | … | … | … |

The ID column is a unique identifier for each microcosm. The treatment column is a categorical variable coding for the treatments (abandoned = worms were present in burrows but removed before the start of the experiment; control = no worms or burrows; artificial = burrows but no worms ever; earthworms = burrows and earthworms throughout the experiment) and is used in the global model. The tunnel column is the dummy variable used to test for the effects of tunnels, with the three treatments that contained burrows coded as 1 and the control coded as 0. The castings column redefines the treatments to test for effects of castings; thus, the abandoned and worm treatments are coded as 1 and the artificial and control treatments are coded as 0. The worm column is a dummy variable to test for the effects of the presence of worms. It has the worm treatment defined as 1 and the other three treatments are 0. The sampling times column has those microcosms sampled only at the end of the experiment coded as 1 and the microcosms sampled at multiple time steps are coded as 0.

**Explanation of K (number of parameters) in our microarthropod abundance analysis (see Table 1. Regression fit statistics for models predicting microarthropod abundance).**

In the null model, K = 2 when Poisson regression is used, with one parameter for the mean and one for the sampling times variable. With negative binomial, the null model has K = 3 because there is an additional parameter to account for overdispersion.

In the tunnels, castings, and worm models, there is a one more parameter than the null because of the dummy variable.

The global or “treatment” model has a further two parameters than the dummy variable models because there are four treatment levels (number of levels – 1).
